# Spatial and temporal in vivo analysis of circulating and sessile immune cells in mosquitoes: hemocyte mitosis following infection

**DOI:** 10.1186/1741-7007-11-55

**Published:** 2013-04-30

**Authors:** Jonas G King, Julián F Hillyer

**Affiliations:** 1 Department of Biological Sciences, Vanderbilt University, VU Station B 35-1634, Nashville, TN 37235, USA

**Keywords:** Hemocyte, Hemocoel, Circulating, Sessile, Hemolymph, Phagocytosis, Immunity, Mosquito, Anopheles gambiae

## Abstract

**Background:**

Mosquitoes respond to infection by mounting immune responses. The primary regulators of these immune responses are cells called hemocytes, which kill pathogens via phagocytosis and via the production of soluble antimicrobial factors. Mosquito hemocytes are circulated throughout the hemocoel (body cavity) by the swift flow of hemolymph (blood), and data show that some hemocytes also exist as sessile cells that are attached to tissues. The purpose of this study was to create a quantitative physical map of hemocyte distribution in the mosquito, *Anopheles gambiae*, and to describe the cellular immune response in an organismal context.

**Results:**

Using correlative imaging methods we found that the number of hemocytes in a mosquito decreases with age, but that regardless of age, approximately 75% of the hemocytes occur in circulation and 25% occur as sessile cells. Infection induces an increase in the number of hemocytes, and tubulin and nuclear staining showed that this increase is primarily due to mitosis and, more specifically, autonomous cell division, by circulating granulocytes. The majority of sessile hemocytes are present on the abdominal wall, although significant numbers of hemocytes are also present in the thorax, head, and several of the appendages. Within the abdominal wall, the areas of highest hemocyte density are the periostial regions (regions surrounding the valves of the heart, or ostia), which are ideal locations for pathogen capture as these are areas of high hemolymph flow.

**Conclusions:**

These data describe the spatial and temporal distribution of mosquito hemocytes, and map the cellular response to infection throughout the hemocoel.

## Background

The insect immune response relies upon innate reactions and involves both cellular and humoral components [1-3]. The immune cells, called hemocytes, exist within an open circulatory cavity called the hemocoel [4,5], where they phagocytose foreign organisms and help coordinate the humoral response to infection [1,3]. In mosquitoes, the immune response reduces the number of pathogens that survive inside the body [6-10] and may either prevent the transmission of disease-causing pathogens or inadvertently maintain vectorial capacity by allowing the insect to survive long enough to transmit an infection. Thus, because of its effect on transmission dynamics, the mosquito immune response could be harnessed for the control of mosquito-borne diseases [11-13].

In both the culicine and anopheline mosquito lineages there are several morphologically distinct classes of hemocytes: granulocytes are involved in the phagocytosis response, oenocytoids are involved in the melanization response, and prohemocytes are small cells of unknown function that have been hypothesized to serve as hematopoietic progenitors [14-16]. While the sub-types of hemocytes that circulate within the mosquito hemocoel are known, basic aspects of hemocyte biology, such as hemocyte numbers, proliferation and spatial distribution, remain poorly understood. For example, aside from a recent study on the interaction between hemocytes and the heart [17], the location, activity and number of sessile hemocytes (hemocytes that exist attached to tissues) remain unknown. Likewise, the number of circulating hemocytes within individual mosquitoes continues to be debated, as similar techniques have sometimes led to markedly dissimilar counts [14,18,19]. Nevertheless, a strong consensus indicates that naïve adult mosquitoes contain between 1,000 and 3,000 circulating hemocytes [14,18,20-22], which is similar to the number of hemocytes present in the related dipteran, *Drosophila melanogaster*[23,24].

In adult mosquitoes and other insects the number of circulating hemocytes changes in response to infection, age and physiological state [18,20,22,24-30]. It has often been hypothesized that this change is due to either a release of sessile hemocytes into circulation, or the replication or differentiation of prohemocytes [14,19,20,24,31]. However, in adult insects little evidence supports these hypotheses, as no hematopoietic organ has been identified in the adult stage [23,32], and although the replication of circulating hemocytes has been visualized in insect larvae [29,33-35], there is a scarcity of direct evidence showing that this also occurs in adults. Nevertheless, in mosquitoes the theory of hemocyte replication is strongly supported by bromodioxyuridine- and thymidine-based studies that have documented hemocyte proliferation somewhere in the body of adult *Aedes* spp. in response to blood feeding or infection with filarial nematodes, respectively [20,21].

In the present study we scrutinize the organization of hemocytes within the entire body of the malaria mosquito, *Anopheles gambiae*, and present the first quantitative hemocyte map of any insect. This map describes the spatial distribution of all hemocytes and shows that approximately three quarters of mosquito hemocytes exist in circulation and one quarter exists as sessile cells. Furthermore, we show that the number of hemocytes decreases with age but increases after an immune challenge, and that the increase in hemocyte numbers following infection is primarily due to mitosis by circulating hemocytes.

## Results

### Circulating and sessile hemocyte numbers: effect of age and infection

**Figure 1:**
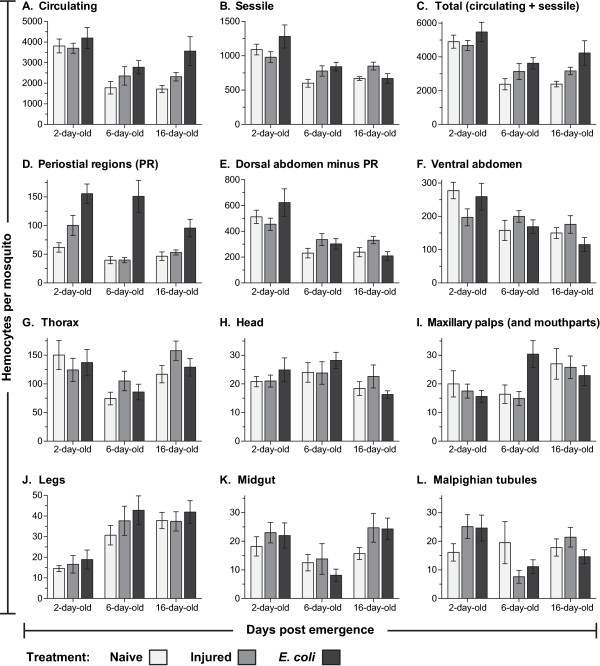
**Systemic hemocyte numbers decrease with age but increase after infection.** Number of circulating (**A**), sessile (**B**) and total (**C**; circulating and sessile) hemocytes at different ages in naïve mosquitoes (light gray), and mosquitoes that had been either injured (medium gray) or infected with *E. coli* (dark gray) for 24 hours. (**D**) Number of sessile hemocytes attached to the dorsal abdominal wall at the location of the ostia (periostial regions). (**E**) Number of sessile hemocytes attached to the dorsal abdominal wall minus the periostial hemocytes. (**F**) Number of sessile hemocytes attached to the ventral abdominal wall. (**G-L**) Number of sessile hemocytes in the thorax (**G**), head (**H**), maxillary palps (**I**), legs (**J**), midgut (**K**), and Malpighian tubules (**L**). Column heights mark the average number of hemocytes per mosquito, and whiskers denote the standard error of the mean.

**Figure 2:**
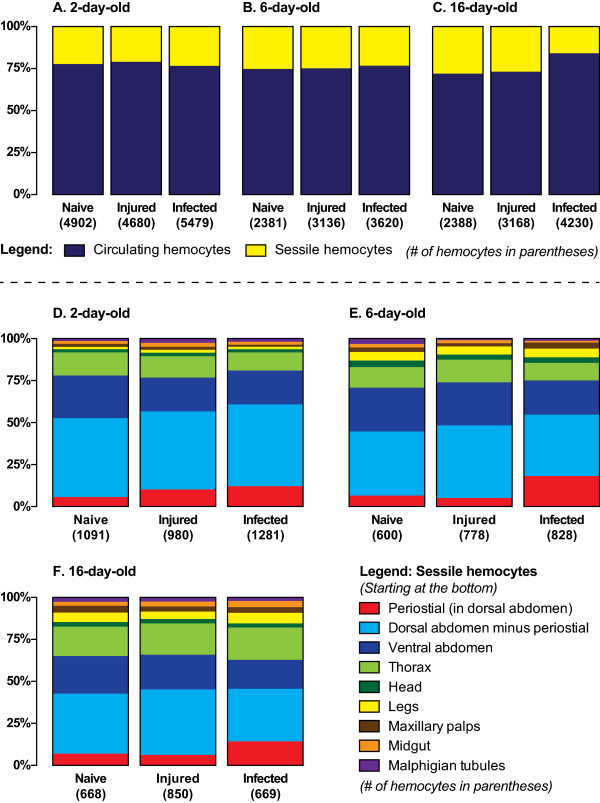
**Proportional distribution of hemocytes throughout the mosquito hemocoel. A**-**C**. Average proportional distribution of circulating and sessile hemocytes in differently-aged naive mosquitoes or mosquitoes that have been subjected to injury or *E. coli* infection for 24 hours. Sessile hemocytes comprise approximately 25% of the total hemocyte population. **D**-**F**. Average proportional distribution of sessile hemocytes throughout the hemocoel in differently-aged naive mosquitoes or mosquitoes that have been subjected to injury or *E. coli* infection for 24 hours. Infection induces a significant increase in the number of periostial hemocytes.

Examination of fluorescently labeled hemocytes revealed that at two days post-eclosion, naïve mosquitoes contain an average of 3,811 circulating hemocytes and that this number decreases to 1,781 and 1,720 by six and sixteen days after emergence, respectively (Figure 1A). At two days post-eclosion, naïve mosquitoes contain an average of 1,091 sessile hemocytes, and this number decreases to 600 and 668 by six and sixteen days after emergence, respectively (Figure 1B). Thus, naïve mosquitoes contain an average of 4,902, 2,381 and 2,388 hemocytes at two, six and sixteen days post-eclosion (Figure 1C). Together, these data signify that the number of hemocytes in naïve mosquitoes drops with age (one way (1 W) analysis of variance (ANOVA) *P* <0.0001) and that, regardless of age, sessile hemocytes comprise approximately 25% of the total hemocyte population (1 W ANOVA *P* = 0.1536; Figure 2A-C).

At six and sixteen days post-eclosion, injury induces a 32% and 33% increase in the total number of hemocytes, respectively, and *E*. *coli* infection induces a 52% and 77% increase in the total number of hemocytes, respectively (Figure 1C). At two days post-eclosion, however, injury and *E*. *coli* infection induce a more modest 5% reduction and 12% increase in the total number of hemocytes, respectively. Statistical comparison of total hemocyte numbers in naïve, injured and *E*. *coli* infected mosquitoes by two-way (2 W) ANOVA showed that, across the three treatment groups, the total number of hemocytes drops with age (*P* <0.0001), which is exemplified by the dramatic reduction that occurs between days two and six post-eclosion. Two-way ANOVA also revealed that treatment affects total hemocyte numbers (*P* = 0.0027), and post-hoc Sidak’s multiple comparisons showed that this is due to a significant infection-induced increase in the total number of hemocytes (*P* = 0.0021). No significant interaction was detected between age and treatment group (*P* = 0.5604).

Independent analysis of circulating and sessile hemocyte numbers revealed that infection has a different effect on these two cell populations (Figures 1A-B and 2A-C). Shared between circulating and sessile hemocytes is that, across all treatment groups, there is an age-specific reduction in the number of hemocytes (2 W ANOVA *P* <0.0001 for both cell populations). However, although treatment does not have a significant effect on the number of sessile hemocytes (2 W ANOVA *P* = 0.1106), treatment induces a change in the number of circulating hemocytes (2 W ANOVA *P* = 0.0043). Sidak’s post-hoc analysis revealed that this treatment-induced change in circulating hemocyte numbers is due to an increase in the number of circulating cells following infection, which is especially pronounced in older mosquitoes (*P* = 0.0037). Finally, no significant interaction was detected between age and treatment group for either the circulating (*P* = 0.4276) or sessile (*P* = 0.0521) hemocyte populations. Together, these data show that the number of circulating and sessile hemocytes dramatically drops several days after eclosion, and that the infection-induced increase in total hemocyte numbers is primarily due to an increase in the number of hemocytes that circulate throughout the hemocoel.

### The majority of sessile hemocytes exist attached to the abdominal epidermis, trachea, and heart-associated periostial regions

**Figure 3:**
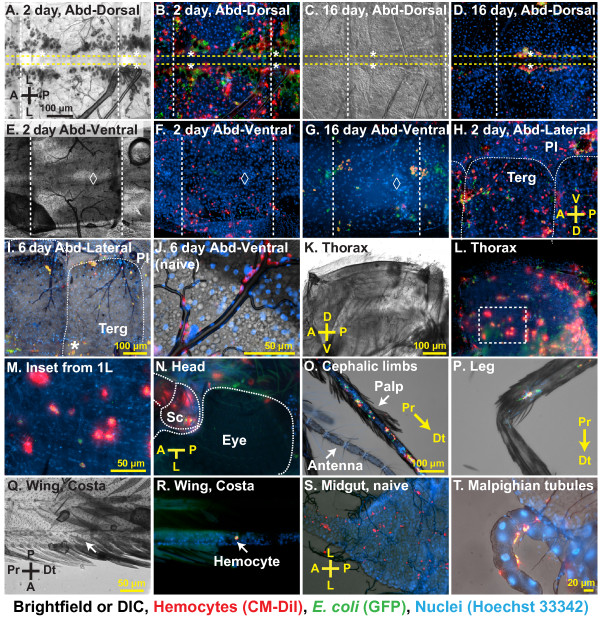
**Sessile hemocytes are phagocytic and are distributed throughout the mosquito body.** (**A-B**) Bright field and fluorescence overlay of sessile hemocytes in the dorsal abdomen of two-day-old mosquitoes at 24 hours post-infection with *E. coli* (red, CM-DiI stained hemocytes; green, GFP-*E. coli*; blue, Hoechst 33342 stained nuclei). Hemocytes are distributed throughout the body wall and melanization products have been internalized by the pericardial cells that flank the heart. Yellow horizontal dotted lines outline the heart, asterisks mark the location of ostia, and white vertical dotted lines mark the abdominal sutures. (**C-D**) DIC and fluorescence overlay of sessile hemocytes in the dorsal abdomen of 16-day-old mosquitoes at 24 hours post-infection with *E. coli*. Hemocytes are aggregated in the periostial regions. (**E-G**) Bright field and fluorescence overlays of sessile hemocytes in the ventral abdomen of two-day-old (**E-F**) and sixteen-day-old (**G**) mosquitoes at 24 hours post-infection with *E. coli*. Diamonds mark the location of the abdominal ganglia. (**H-J**) Bright field and fluorescence overlays of sessile hemocytes adhered to the abdominal wall and the tracheae in the abdominal pleuron (Terg, tergite; Pl, pleurite). (**K-M**) Bright field and fluorescence overlays of sessile hemocytes in the thoracic indirect flight muscles. The box in panel **L** is magnified in panel **M**, and shows hemocytes as well as rows of densely packed myocyte nuclei. (**N-R**) Overlays of hemocytes in the head (**N**; Sc, scape), maxillary palps (**O**), legs (**P**), and wings (**Q-R**). (**S-T**) Overlays of hemocytes bound to the midgut (**S**) and Malpighian tubules (**T**). Unless otherwise stated, samples shown are from six-day-old adults at 24 hours post-infection with *E. coli*. Orientation guides and scale bars apply to the image they appear in and each subsequent image, until new guides are presented. A, anterior; P, posterior; D, dorsal; V, ventral; L, lateral; Pr, proximal; Dt, distal.

While a considerable amount of work has investigated the biology of mosquito circulating hemocytes, the location and activity of sessile hemocytes has received little or no attention. Systemic analysis of sessile hemocytes in *A*. *gambiae* revealed that they are consistently attached to the thoracic and abdominal cuticular epidermis, the visceral organs, and inside all appendages, with the exception of the antennae and halteres (Figures 2D-F and 3). The anatomical location that contains the vast majority of sessile hemocytes is the abdominal wall, which in two-, six- and sixteen-day-old naïve mosquitoes contains an average of 851, 429 and 435 hemocytes (Figures 1D-F, 2D-F and 3A-J), indicating that aging results in a 50% reduction in the number of abdominal sessile hemocytes (1 W ANOVA *P* <0.0001). In relation to the entire body, abdominal sessile hemocytes represent 78%, 71% and 65% of the total sessile hemocyte population at two, six and sixteen days post-eclosion, respectively.

In naïve mosquitoes of all ages, approximately two thirds of the abdominal sessile hemocytes are located in the dorsal abdomen and one-third in the ventral abdomen (Figures 2D-F and 3A-J). Of the dorsal abdominal hemocytes, between 11% and 16% are present in the periostial regions (Figures 1D, 2D-F and 3A-D; regions surrounding the valves of the heart, or ostia; [17]) and, in both the dorsal and ventral abdomen, a large proportion of abdominal sessile hemocytes exist attached to the respiratory trachea (Figure 3H-J). While the ratio of dorsal versus ventral hemocytes is similar for all age groups, their spatial distribution changes with age. Specifically, abdominal sessile hemocytes are more widely dispersed in younger insects when compared to older insects (compare Figure 3B to D, and 3F to G).

In response to infection, the vast majority of abdominal sessile hemocytes phagocytose GFP-expressing *E*. *coli*, indicating that their ability to sequester pathogens is similar to that of circulating hemocytes (Figure 3). However, at all ages tested, injury or infection did not induce changes in abdominal sessile hemocyte numbers with one notable exception: the periostial regions. Specifically, infection for 24 hours led to a 251%, 377% and 204% increase in the number of periostial hemocytes in two-, six-, and sixteen-day-old mosquitoes (Figures 1D, 2D-F; 2W ANOVA *P* <0.0001), numbers that are in agreement with our recent report detailing the physiological interaction between the mosquito circulatory and immune systems [17]. In two-day-old mosquitoes, the increase in periostial hemocyte numbers was commonly coupled with the presence of melanization products inside the pericardial cells that line the heart (dark matter in Figure 3A, compare to 3C).

### Sessile hemocytes exist attached to the indirect flight muscles and thoracic cuticular epidermis

A large proportion of the thorax is composed of the indirect flight muscles, and sessile hemocytes were found distributed throughout them with no discernible pattern (Figure 3K-M). Light refraction by the myofibers prevented high resolution imaging of thoracic sessile hemocytes, but most of these cells had clearly phagocytosed bacteria following infection. While these cells were immunologically active, injury or infection did not impact thoracic hemocyte numbers (Figure 1G; 2W ANOVA *P* = 0.5555). Aging, on the other hand, impacted thoracic hemocyte numbers (2W ANOVA *P* = 0.0016): two- and sixteen-day old mosquitoes contained more thoracic hemocytes than six-day-old mosquitoes (Sidak’s *P* ≤0.0070 for both). Overall, the number of thoracic hemocytes averaged 114 and 117 in naïve and infected mosquitoes, respectively, or 14% and 13% of the total number of sessile hemocytes (Figures 1G and 2D-F).

### Sessile hemocytes exist inside the head, maxillary palps and legs

Sessile hemocytes are present in all major appendages, except for the antennae and halteres. Hemocytes were present in the head, maxillary palps and legs of all individuals, and hemocytes in all of these locations were highly phagocytic (Figure 3N-P). Regardless of age or treatment, mosquitoes had approximately 20 hemocytes in the head, and these were most commonly found near the neck and around the base of each antenna (Figures 1H and 3N). The maxillary palps also contained approximately 20 hemocytes, which in two-day-old mosquitoes were most commonly located near the base of these sensory appendages while in older mosquitoes were randomly distributed across their entire length (Figures 1I and 3O). Finally, all mosquitoes contained hemocytes in their legs, but the number of cells in these appendages dramatically changed with age (2W ANOVA *P* <0.0001): the legs of naïve mosquitoes at two, six and sixteen days post-eclosion contained an average of 15, 31 and 38 hemocytes, respectively (Figures 1J and 3P). This age-associated doubling of leg hemocytes is even more pronounced when considering that the total number of sessile and circulating hemocytes decreases with age (Figure 1A-B). Finally, injury or infection had no effect on the number of hemocytes inside the head, palps, and legs (2W ANOVA *P* ≥0.2542 for all).

Few hemocytes were observed inside the wings: only 17%, 10% and 26% of two-, six- and sixteen-day-old mosquitoes contained hemocytes inside the wings, and when observed, the average number of cells was <3.25 per mosquito (Figure 3Q-R). Also, while hemocytes were observed at the base of the antennae and halteres, they were not observed inside of these appendages (Figure 3N-O).

### Small numbers of hemocytes exist attached to the midgut and Malpighian tubules

The midgut and Malpighian tubules had a small but variable number of sessile hemocytes, and no clear pattern was observed between any age or treatment groups (Figures 1K-L and 3S-T). An average of 20 hemocytes per mosquito were scattered along the basal surface of both of these two organs, and a high proportion of these hemocytes were phagocytic.

### Hemocytes may be involved in developmental processes

**Figure 4:**
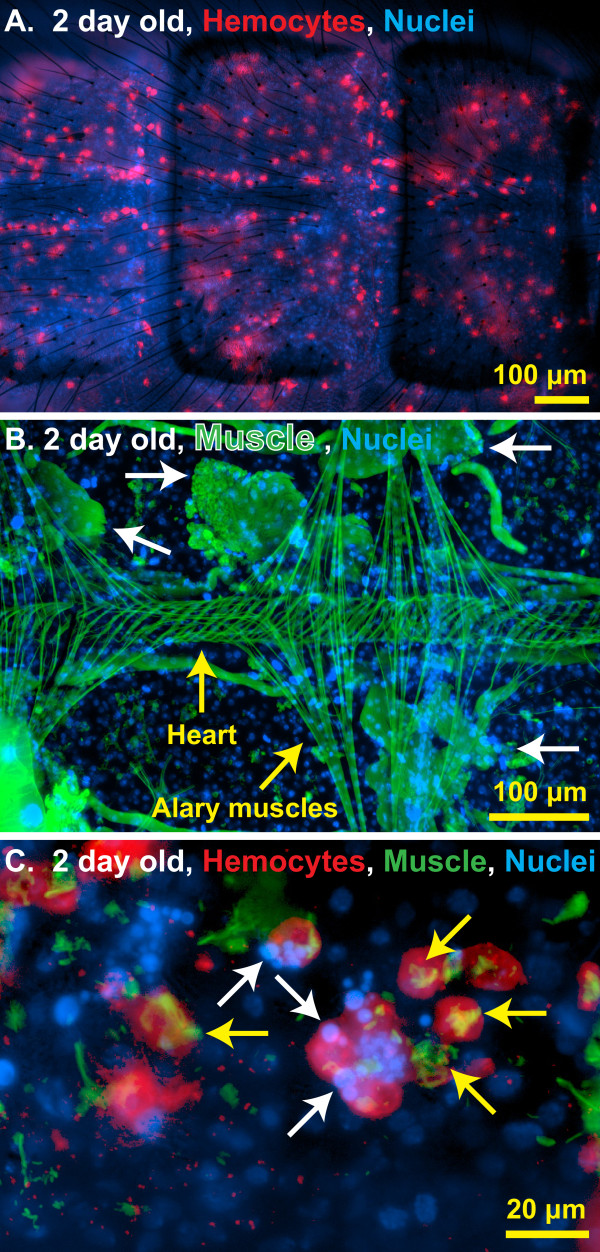
**Sessile hemocytes interact with autolysing swimming muscles.** (**A**) Fluorescence overlay showing hemocytes (red, CM-DiI) evenly distributed throughout a portion of the dorsal abdominal wall of a two-day-old naïve mosquito. DNA is stained blue with Hoechst 33342. (**B**) Fluorescence overlay of a portion of the dorsal abdomen of a two-day-old naïve mosquito showing the heart, the alary muscles, and the remnants of larval swimming muscles undergoing autolysis (white arrows). Muscle is stained green with AlexaFluor-488-conjugated phalloidin and DNA is stained blue with Hoechst 33342. (**C**) Higher magnification fluorescence overlay of hemocytes (red) engaged in an apparent interaction with the condensed nuclear material (blue; for example, arrows) of autolysed abdominal myocytes (green; for example, yellow arrows) in a two-day-old naïve mosquito.

Mosquito hemocytes have largely been studied because of their role in immunity. However, a series of labeling experiments suggest that they may also be involved in development. First, *in vivo* hemocyte staining showed that abdominal sessile hemocytes are more prevalent in newly emerged mosquitoes when compared to older mosquitoes, and that in young mosquitoes they are evenly dispersed throughout the abdominal wall (Figures 1D-F, 3A-G and 4A). Second, muscle staining revealed that at one to two days post-emergence, the histolysis of larval swimming muscles has not been completed (Figure 4B). Finally, co-staining of hemocytes and muscle revealed that at one to two days post-emergence some of the abdominal sessile hemocytes had internalized muscle fibers and pyknotic nuclear materials that were likely remnants of the larval swimming muscles (Figure 4C). Muscle degradation by sessile hemocytes was not observed at days six and sixteen post-emergence, a time when all larval swimming muscles had been completely broken down. Thus, hemocytes may be involved in shaping the internal architecture of adult mosquitoes during the first few days after eclosion.

### Infection induces mitosis in circulating hemocytes

**Figure 5:**
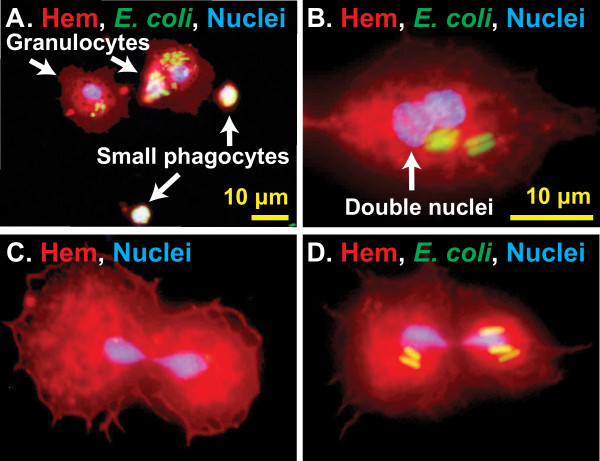
**CM-DiI staining suggests hemocytes replicate in circulation.** (**A**) Fluorescence overlay showing small phagocytes (approximately 5 μm diameter), commonly referred to as prohemocytes, alongside typical granulocytes. Hemocytes (Hem) were stained red with CM-DiI, DNA blue with Hoechst 33342, and GFP-*E. coli* is green. White color signifies overlap of all three fluorescent channels, indicating a high level of phagocytosis. (**B-D**) Fluorescence overlays showing granulocytes with nuclei that appear to be fused or dividing, suggesting that they are undergoing autonomous cell division. Some of these hemocytes had phagocytosed *E. coli*.

During this study we observed that infection induces an increase in hemocyte numbers. The increase in hemocyte numbers is due to an increase in circulating cells, and visual examination of CM-DiI/Hoechst 33342 stained hemocytes suggested that a small proportion of circulating cells were undergoing cell division. For example, some cells contained two nuclei, and the two nuclei at times appeared to be interconnected (Figure 5A-D). To test whether hemocytes divide in circulation, we exposed hemocytes to compounds that delay or halt mitosis and directly measured cell replication using tubulin and nuclear staining.

**Figure 6:**
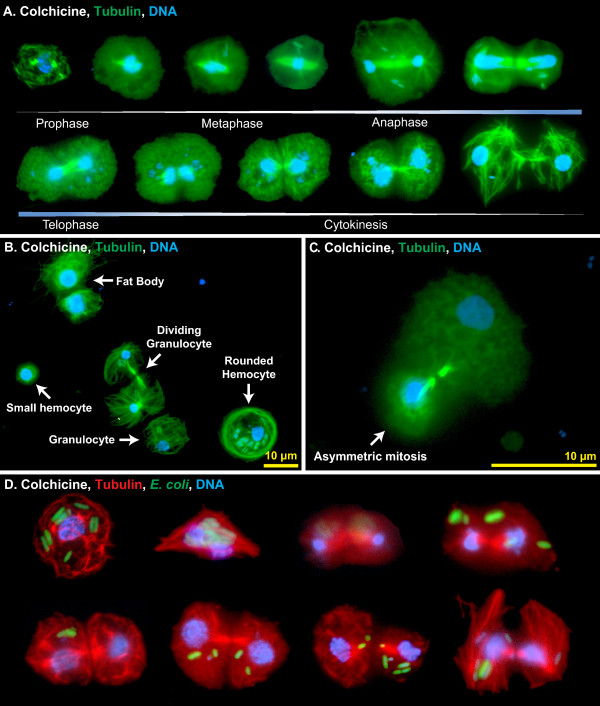
**Hemocytes undergo mitosis in the hemocoel.** (**A**) Fluorescence overlay montage of perfused hemocytes from six-day-old mosquitoes showing all stages of mitosis. Mosquitoes had been treated with colchicine prior to perfusion, tubulin is stained green, and DNA is stained blue. (**B**) Fluorescence overlay of tubulin-stained (green) perfused cells from an *E. coli*-infected mosquito, showing differences in cytoskeletal arrangements. Note the crosshatched cytoskeleton in the granulocyte and smaller hemocyte and the rounded tubulin border in the ‘rounded’ hemocyte. The mitotic bodies and reordered cytoskeleton of a dividing granulocyte can be seen at center. Two fat body cells are also present and they contain larger nuclei and a more unstructured tubulin cytoskeleton when compared to a granolucyte. (**C**) Asymmetric mitosis of hemocytes resulting in one small daughter cell (bottom) and a granulocyte-sized daughter cell (top). (**D**) Fluorescence overlay montage of perfused hemocytes from mosquitoes infected with *E. coli*, showing that immune activated and phagocytic hemocytes can undergo mitosis. In this montage, tubulin is stained red, GFP-*E. coli* are green, and DNA is stained blue with Hoechst 33342.

As an initial assay, mosquitoes were treated with colchicine, and hemocytes were then perfused, fixed, and labeled with anti-tubulin antibody and Hoechst 33342. Because colchicine binds tubulin and interferes with microtubule polymerization, thus slowing down mitosis and enriching the number of mitotic cells, we reasoned that if hemocyte replication were occurring then treatment with this chemical would allow for the direct visualization of mitotic events. Indeed, a small percentage of hemocytes were observed undergoing mitosis, with the mitotic stages of prophase, metaphase, anaphase and telophase, as well as cytokinesis, all represented (Figure 6A). Most mitotic events led to two daughter cells of similar size (Figure 6A-B, D). However, grossly asymmetric cytokinesis was also observed (Figure 6C). The smaller cells resulting from these asymmetric divisions were similar in size to the small hemocytes commonly referred to as prohemocytes (see Figures 5A and 6B), and together with the observation that these smaller cells are phagocytic, these data suggest that what has been previously called a prohemocyte could represent a small form of a granulocyte. Finally, the vast majority of hemocytes that were undergoing mitosis had phagocytosed bacteria, indicating that replication is induced in immunologically active cells (Figure 6D).

**Figure 7:**
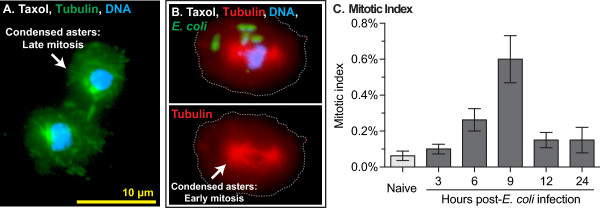
**Infection induces mitosis in circulating hemocytes.** (**A-B**) Taxol treatment of hemocytes results in spindle contraction and highly condensed asters. In panel A, tubulin is stained green and DNA is stained blue. In panel B, tubulin is stained red, GFP-*E. coli* are green, DNA is stained blue, and dotted lines denote the cell boundary. Triple fluorescence samples such as the ones seen in B were used to quantify mitotic indices. (**C**) Mitotic indices in naïve mosquitoes and infected mosquitoes at different times following immune challenge. Proliferation peaks at nine hours post-infection. Column heights mark the average and whiskers denote the standard error of the mean.

While the detection of any mitotic cell in a static tissue is often considered as a sign of considerable cellular proliferation, we set out to measure more accurately the rate of hemocyte mitosis. To quantify mitotic events, hemocytes were treated with taxol, a chemical that stabilizes microtubules, leading to the arrest of mitosis and the production of highly condensed microtubule asters. As expected, taxol treatment confirmed that circulating hemocytes undergo replication and showed that the rate of replication increases in response to infection (1W ANOVA *P* <0.0001; Figure 7A-C). Specifically, 0.05% of circulating hemocytes from five-day-old naïve mosquitoes were observed undergoing mitosis. This mitotic index increased to 0.63% by nine hours post *E*. *coli* infection and then dropped to around 0.1% by 12 and 24 hours post-infection. At least one mitotic cell was observed in (1) half of the naïve mosquitoes examined, (2) 100% of the infected mosquitoes assayed at six and nine hours post-infection, and (3) 60% to 70% of infected mosquitoes assayed at the other time points. The average mitotic index of all samples collected after infection was 0.24%. Assuming that the process of hemocyte mitosis in mosquitoes occurs at roughly the same speed as mitosis in hemocyte-like *Drosophila* S2 cells (20 minutes; [36]), autonomous cell division in mosquitoes accounts for an approximately 18% increase in hemocyte numbers by 24 hours of infection. Considering that our method only allowed for the reliable identification of mitotic cells from pro-metaphase onward, we estimate that the actual increase in hemocyte numbers approaches 25% per day.

### Cytoskeleton rearrangement occurs during hemocyte-mediated immune responses

While examining hemocytes for mitotic events, we noticed that arrangement of microtubules was different in a small proportion of the larger phagocytic cells when compared to the smaller phagocytic cells, suggesting that cytoskeleton dynamics are important during the phagocytosis response. Specifically, some large and heavily phagocytic hemocytes were more rounded and contained a dense ring of tubulin around their margins (Figure 6B, ‘rounded hemocyte’), whereas most hemocytes (≤10 μm in diameter) from naïve and infected mosquitoes had a cytoskeleton that was crosshatched or radial in appearance (Figure 6B). Perhaps hemocytes with dense tubulin rings are those that are engaging in the ‘pooled’ phagocytosis process previously described in *Aedes aegypti*[22,37].

## Discussion

Insects lack adaptive (acquired) immunity, as classically defined [38]. However, with an innate immune system they have filled virtually every imaginable terrestrial and freshwater niche, and by many measures have become the most successful group of multicellular organisms [39,40]. The insect immune system is composed of both cellular and humoral factors and relies upon the actions of several types of immune cells called hemocytes [32]. These cells exist within an open circulatory cavity [4,5], where they phagocytose and encapsulate foreign elements and help coordinate the humoral response to infection [16,17,41]. Because insects are of paramount economic and medical importance, it is surprising that major gaps still exist in our basic knowledge of hemocyte biology. Among these gaps are the numbers of hemocytes present in mosquitoes, as well as their spatial distribution. Specifically, the number of circulating hemocytes in mosquitoes continues to be debated [18-20,42], and until this study, the spatial distribution of sessile hemocytes within the hemocoel had not been addressed.

Aging and immune stimuli are known to impact circulating hemocyte numbers in multiple insect species [23,24,27,29,30,43], including mosquitoes [14,18,20-22,42]. It has often been assumed that the release of hemocytes from a discrete hematopoietic organ, or the replication of a progenitor cell type known as the prohemocyte, leads to increases in circulating hemocyte numbers. However, no hematopoietic organ has been found in any adult insect, the release of sessile hemocytes into circulation has not been reported in this life stage and there is a scarcity of data on the replication of circulating hemocytes in adult insects [32]. Using novel techniques, we herein present the first quantitative map of hemocyte distribution in any insect. Along with describing the spatial increases in hemocyte numbers following infection and the decreases in hemocyte numbers associated with aging, we also report direct evidence of mitosis by mosquito circulating hemocytes.

A variety of techniques have been employed to study hemocyte biology. Hemocytes are usually collected from circulating populations by some variation of hemolymph perfusion or extraction [14,21,22], and most prior studies have relied on *ex vivo* examination of live or fixed cells, although flow cytometry has also been used [44,45]. *In vivo* studies have been conducted in larval *Drosophila*[24,46], but with one exception [17], no direct *in vivo* examination of hemocytes has been conducted in non-drosophilids. Here, using CM-DiI to stain hemocytes *in vivo* we quantified the number of circulating hemocytes and the distribution and numbers of sessile hemocytes within the entire mosquito. We found that sessile hemocytes form a substantial proportion (about 25%) of the total hemocyte population and are heavily phagocytic. Many of the sessile hemocytes in the abdomen, the compartment of the hemocoel where they are most abundant, are found attached to the trachea or near the ostia of the heart (periostial regions). We hypothesize that aggregation near the ostia and trachea represents an adaptation for increased immune surveillance, as this places hemocytes in areas of high hemolymph flow and in areas of potential pathogen entry, respectively. Indeed, immune factors are transcribed in heart-associated tissues [47], and we hypothesize that these factors are produced by immunologically active periostial hemocytes (not the pericardial cells) [17]. Finally, in addition to being a strategic site for pathogen capture, hemocyte positioning at tracheal sites could also enhance their oxygen supply, as has been hypothesized in lepidopteran larvae [48].

Total hemocyte numbers decline over the first six days of an adult mosquito’s life, as had been previously shown in the circulating hemocytes of *Aedes aegypti* and *Anopheles gambiae*[14,22]. The reason for this decline is not clear, but it may be related to development. Specifically, in *D*. *melanogaster* hemocytes are involved in the digestion of apoptotic cells during ecdysis-associated tissue remodeling [49], a finding that is consistent with our observation that hemocytes degrade larval swimming muscles following eclosion. In *D*. *melanogaster* hemocytes are known to originate from two distinct cell lineages [50]. In larvae, embryonic hemocytes occur as immunologically active sentinel cells, while lymph gland derived hemocytes are deployed following an immune challenge [51] and, presumably, during the process of lymph gland degeneration that occurs during eclosion. We hypothesize that the dramatic age-specific decline in hemocyte numbers seen in this study could be due to lineage-specific hemocyte apoptosis following the completion of ecdysis-associated tissue remodeling. Circulating hemocyte counts in *A*. *gambiae* indirectly support this hypothesis, as pupae contain considerably more hemocytes than three-day-old adults [14].

Multiple studies have reported that circulating hemocytes increase in number following immune stimulation [14,18,20,21,26]. Our circulating hemocyte data are in agreement with these findings, but our data on sessile hemocytes show that the only locations where infection induces a consistent and significant increase in sessile hemocytes are the periostial regions. We have previously shown that this increase in periostial hemocytes is due to the adhesive capture and subsequent migration of circulating hemocytes [17], which suggests that hemocyte replication in adult mosquitoes occurs in circulation. In the present study, tubulin staining of hemocytes showed that a small proportion of circulating hemocytes continuously undergo mitosis, and that the proportion of mitotic hemocytes increases following infection. As stated by Wieder [52], tubulin-based mitotic assays represent the ‘one true direct measure of cellular proliferation’, although this procedure often underestimates the rate of mitosis. Therefore, the mitotic indices reported here should be viewed as a conservative measure of mitosis. Regardless, our results suggest that hemocytes are capable of proliferating by autonomous division. Furthermore, because all phases of mitosis are observed in circulation, the data strongly suggest that the increases in circulating hemocyte numbers following infection are primarily due to mitosis by already circulating cells. Finally, based on the likely division time of hemocytes (20 minutes; [36]) and our measured mitotic indices, proliferation of circulating hemocytes in infected mosquitoes explains a substantial proportion of the proliferation seen in our systemic measures. Whether replication also occurs within the sessile hemocyte population remains unknown, but no hematopoietic organ was identified during the course of this study.

In adult insects it has been hypothesized that new hemocytes arise from the replication and differentiation of a hemocyte morphotype known as the prohemocyte [3]. Hemocytes matching this physical description (≤5 μm in diameter and possessing a high nuclear to cytoplasm ratio) were commonly seen during this study. However, our observation of these prohemocytes ‘budding’ from larger ones (asymmetric cytokinesis) suggests that this hypothesis is incorrect and, furthermore, that the opposite might be true: we hypothesize that small granulocytes (≤5 μm) that are immunologically active (phagocytic) are produced from mature granulocytes (approximately 10 μm). The phagocytic nature of the small hemocytes observed in this study is in conflict with the classical definition of prohemocytes [53], but our finding is loosely in agreement with the view that prohemocytes are not multipotent stem cells but are instead fate restricted [51,54]. Ultrastructural evidence supports our hypothesis by showing that the cell types are very similar in subcellular structure [26]. Furthermore, hemocytes are bathed in a nutrient rich medium and are known to increase in size upon immune stimulus [16,55,56]. Therefore, it would not be surprising if these smaller daughter cells are produced with a minimum of sacrifice to the mother cell but are able to mature into capable immune cells in a relatively short period of time. Recent data from mammalian systems show that asymmetric partitioning of cellular components between daughter cells is common and purposeful [57]. The authors propose that certain types of asymmetric mitosis might represent a physiological mechanism for producing a ‘pristine’ daughter cell from a mother cell that contains abundant waste. We speculate that asymmetric mitosis could play a role in maintaining a healthy hemocyte population following a phagocytic response to infection.

## Conclusion

In conclusion, the data presented herein represent three advancements in our understanding of insect hemocyte biology. First, by creating the first insect quantitative hemocyte map, we describe the anatomical distribution of hemocytes and show that sessile hemocytes form a major component of the mosquito immune system. Second, by performing qualitative and quantitative analyses in different physiological states, we show that the number of hemocytes changes significantly with age and in response to infection. Lastly, we present direct evidence of mitosis in the circulating hemocytes of an adult insect, showing that hemocyte proliferation in adult mosquitoes does not require a discrete organ or progenitor cell type.

## Methods

### Mosquito rearing and maintenance

*Anopheles gambiae* (G3 strain) were reared and maintained as described [5]. Briefly, larvae were hatched in deionized water in plastic containers and fed a blend of koi food and yeast. Pupae were separated by size, allowed to emerge into adults in plastic buckets, and fed a 10% sucrose solution *ad libitum*. Rearing and maintenance was done in an environmental chamber at 27°C, 75% relative humidity and a 12 hour light/12 hour dark photoperiod. All experiments carried out during this study were in compliance with Vanderbilt University's ethical guidelines.

### Mosquito injections and bacterial infections

For injections, mosquitoes were cold-anesthetized and a finely pulled glass needle was shallowly inserted into the thoracic anepisternal cleft. A volume of 0.2 μl was injected into the hemocoel and mosquitoes were then returned to an environmental chamber until assayed.

For bacterial infections, GFP-expressing *Escherichia coli* (modified DH5α) were grown overnight at 37°C in Luria-Bertani’s rich nutrient medium (LB broth), and cultures were normalized to OD_600_ = 4 using a BioPhotometer plus spectrophotometer (Eppendorf AG, Hamburg, Germany) prior to being injected into mosquitoes. To quantify the infection dose precisely, dilutions of OD_600_ = 4 *E*. *coli* cultures were plated on LB agar with tetracycline, incubated at 37°C overnight, and the colony forming units were counted 18 hours later. On average, OD_600_ = 4 represented an infection dose of 103,000 (+/− 29,000 SD) bacteria per mosquito.

### Hemocyte labeling, visualization and counts

Hemocyte visualization and counts were performed in naïve, injured (injected with sterile LB broth) and *E*. *coli* infected female mosquitoes. Mosquitoes were injured or infected at one, five and fifteen days post-eclosion, and hemocytes were imaged 24 hours post-treatment. Thus, hemocyte imaging and counting for all three groups was performed at two, six and sixteen days post-eclosion.

A sequence of procedures, performed in an exact series, was used to analyze systemic hemocyte numbers in individual mosquitoes. Briefly, hemocytes inside a live mosquito were labeled with Vybrant CM-DiI, extracted from the hemocoel by perfusion, and allowed to adhere to a glass slide. While perfused cells were adhering, the mosquito body was aldehyde-fixed and dissected, and the carcass and internal organs were mounted on glass slides. Finally, the perfused hemocytes were then aldehyde-fixed and mounted on a glass slide. After preparing these slides, the sessile (attached to the carcass or internal organs) and circulating (perfused) hemocytes were counted. Sessile hemocyte preparations were always counted within two hours of tissue collection. Perfused hemocytes were counted within three days of collection, as unlike carcass and internal organ preparations, these slides could be preserved for several days. For each age group, three treatments were performed (naïve, injured and *E*. *coli* infected), and for each treatment, hemocytes from 10 individual mosquitoes that originated from 10 independent but paired cohorts were examined (one mosquito per treatment per cohort). Each step in this sequence of procedures will now be presented in detail.

*In vivo* hemocyte staining was achieved using the hemocyte staining dye CM-DiI as we have described [17]. Briefly, each live female mosquito was injected with 0.2 μl of a freshly prepared solution consisting of 75 μM CM-DiI (hemocyte stain; Vybrant CM-DiI Cell-Labeling Solution, Invitrogen, Carlsbad, CA, USA) and 0.75 mM Hoechst 33342 (nuclear stain; Invitrogen) in PBS that was warmed to 25°C. After CM-DiI injection, mosquitoes were immediately returned to 27°C for 20 minutes, and then hemolymph was collected.

Circulating cells were collected by perfusing the hemolymph [15,21]. For this, an incision was made through the lateral edge of the eighth abdominal segment using a feather blade and the mosquito was held vertically on a vacuum restraint with the abdomen pointing downwards. A glass microinjection needle was then inserted into the mosquito’s cervical membrane, 200 μL of Schneider's *Drosophila* Medium was injected, and the diluted hemolymph that exited the posterior abdomen was collected onto the center of two 1-cm diameter etched rings on Rite-On glass slides (Gold Seal; Portsmouth, NH, USA). Perfusion was done at a rate of 20 seconds per mosquito, with the first 100 μL collected in one etched ring and the second in the other. Cells were allowed to adhere to the slide for 20 minutes at room temperature, fixed for 10 minutes with 4% paraformaldehyde in PBS, washed three times for five minutes with PBS, and coverslips were mounted with Aqua Poly/Mount (Polysciences; Warrington, PA, USA).

Immediately following perfusion, a 16% paraformaldehyde solution was intrathoracically injected into the mosquito, and the carcass was allowed to fix for 10 minutes. The mosquito was then briefly immersed in 0.2% Tween 20 in PBS, transferred to PBS without Tween, and a cracked feather blade was used to (1) separate the ventral and dorsal portions of the abdomen along the ventral pleural suture (dissection along a coronal plane), and (2) separate the thorax from the abdomen. The midgut and Malpighian tubules were then extracted using 0.2 mm diameter minuten pins and the legs and wings were cut from the body. The thorax was cut in half along a sagittal plane and the head and cephalic appendages were detached as a single unit. All disarticulated mosquito fragments were then mounted under coverslips using Aqua Poly/Mount.

Visual examination and imaging of hemocytes was conducted using a Nikon® 90i compound microscope (Nikon; Tokyo, Japan) equipped with a Nikon® Intensilight C-HGFI fluorescence illumination unit and Photometrics CoolSNAP HQ^2^ (Roper Scientific; Ottobrunn, Germany) and Nikon DS-Qi1Mc digital cameras. Nikon’s Advanced Research NIS-Elements software was used for on-screen viewing and image acquisition. Specimens were viewed under differential interference contrast (DIC), bright field, and/or epi-fluorescence illumination, and Z-stack images were captured using a linear encoded Z-motor and NIS-Elements. To produce two-dimensional images, image stacks were combined to form a focused image using the Extended Depth of Focus (EDF) module of NIS Elements.

Sessile hemocyte counts were conducted on the abdomen, thorax, head, maxillary palps (and other mouthparts), wings, legs, midgut, Malpighian tubules, antennae and halteres. Hemocytes attached to internal tissues were counted through the oculars using 400x-1,000x magnification. Hemocytes inside the head, palps, wings and legs were counted through the transparent cuticle using 400x magnification. When high cell densities were present on large pieces of tissue, the accuracy of ocular counts was confirmed by acquiring 200x or 400x magnification digital images and re-counting the hemocytes using the manual particle counting feature of NIS Elements. Cells were counted as hemocytes if they were labeled with both CM-DiI and Hoechst 33342. In the dorsal abdomen, cells were counted as periostial hemocytes if they were attached to the dorsal vessel at the ostia, or formed part of a contiguous mass of hemocytes that were attached to this region [17]. Circulating (perfused) hemocytes were counted and imaged using 1,000x magnification.

Because background staining was common on the side of the thorax where CM-DiI was injected, thoracic hemocytes were only counted on the side opposite of the injection and this number was doubled to extrapolate hemocyte numbers for the entire thorax. A small parallel study validated this method: when CM-DiI was injected into the abdomens of naïve, injured or infected mosquitoes and hemocytes were counted on both sides of the thorax, values were similar to when hemocyte numbers were calculated by extrapolating from unilateral counts.

Statistical analyses of hemocyte counts were performed by ANOVA. Comparisons that involved one variable (for example, the effect of age on hemocyte numbers in naïve mosquitoes) were performed by one-way ANOVA. Comparisons that involved two variables (for example, the effect of age on sessile hemocyte numbers in naïve, injured and infected mosquitoes) were performed by two-way ANOVA. This latter test yields three distinct *P*-values, which in the case of this study address the questions of (1) whether mosquito age affects the results, (2) whether treatment (naïve, injured, infected) affects the results, and (3) whether treatment has a different effect at different ages and *vice versa* (interaction). When significance by two-way ANOVA was detected (*P* <0.05), pre-planned *post-hoc* comparisons were performed using Sidak’s test.

To confirm the efficiency of our hemolymph perfusion method we performed three independent control experiments. First, analysis of hemocyte counts in the two etched circles for each mosquito revealed that 89% of the hemocytes were collected within the first 100 μl of perfusate and 11% were collected within the second 100 μl of perfusate. The low number of cells collected in the second circle suggests that virtually all of the circulating hemocytes were collected by this method. Second, examination of non-adherent material in the perfusate identified a negligible number of cells per mosquito (<10 cells that stained with DiI and Hoechst 33342), suggesting that nearly all hemocytes are adherent. Third, dorsal preparations of mosquitoes that had been perfused and dorsal preparations of mosquitoes that had not been perfused contained similar numbers of periostial hemocytes, indicating that perfusion does not dislodge sessile hemocytes.

Finally, throughout this study, mosquitoes were discarded if (1) fewer than 90% to 95% of a subsample of perfused hemocytes had incorporated the CM-DiI stain, (2) background staining was obtrusive in whole-mount preparations, or (3) any of the dissected tissues could not be counted (for example, a problem with the dissection). Because maintaining paired cohorts of naïve, injured and infected mosquitoes was a priority, removal of one mosquito from the study resulted in the removal of the entire cohort.

### Co-labeling of hemocytes and abdominal musculature

To visualize the potential interaction between hemocytes and muscle following eclosion, CM-DiI and muscle co-staining was performed in two-day-old mosquitoes. Muscle staining was performed by injecting a solution containing formaldehyde, phalloidin-AlexaFluor-488, Hoechst 33342, and Triton X-100 as described [5].

### Mitosis and mitotic index

Mitosis was directly detected using immunocytochemical staining of tubulin along with Hoechst 33342 nuclear staining. To enhance our ability to detect hemocytes undergoing mitosis, mosquitoes were treated with 10 nM, 100 nM, 1 μM, 10 μM and 100 μM of taxol or colchicine, or 20 μM of MG-132 (Acros Organics; Geel, Belgium) as part of a pilot study. For both taxol and colchicine, a concentration of 1 μM was deemed optimal and used for the visualization and quantization of hemocyte mitotic events. MG-132 was found ineffective, suggesting that the hemocyte spindle checkpoint may be atypical or absent [58].

For qualitative studies, hemocyte mitosis was slowed with colchicine, as this compound enriches the number of mitotic cells, making the spindle bodies ideal for the interpretation of mitotic stages [52]. Here, naïve mosquitoes, or mosquitoes at 3, 6, 9, 12 and 24 hours after infection with *E*. *coli* were injected with 0.2 μL of 1 μM colchicine. Mosquitoes were kept at 27°C for one hour, and the hemocytes were then perfused onto glass slides using 10 μL of 1 μM colchicine in Schneider’s medium. Cells were allowed to adhere to the slides for 20 minutes at room temperature, aldehyde-fixed as above, permeabilized by adding 0.5% Triton X-100 for 5 minutes, and rinsed 3 × 5 minutes with cold PBS. Samples were then blocked with 5% fetal bovine serum (FBS) in PBS for one hour at room temperature, and mouse anti-tubulin antibody (4 μg/mL; Sigma, St. Louis, MO, USA) was applied for one hour at 25°C in blocking solution. Three five-minute washes with PBS were then performed before incubation in 4 μg/mL AlexaFluor-568 goat-anti-mouse antibody (Invitrogen) or Cy2 goat-anti-mouse antibody (Invitrogen) in 5% FBS for one hour at 25°C. Slides were then incubated in 30 μg/mL Hoechst 33342 for 10 minutes, washed 3 × 5 minutes with PBS, and coverslips were mounted using Aqua Poly/Mount.

For quantitative studies, taxol was administered during perfusion as this compound rapidly arrests mitosis with relatively low cytotoxicity, resulting in highly condensed asters that are more confidently recognized than those produced by other drugs [52]. Here, hemocytes were perfused with 10 μL of Schneider’s medium onto an etched ring containing a taxol solution that was instantly diluted to a final concentration of 1 μM. Hemocytes were stained with anti-tubulin and Hoechst 33342 as above.

Mitotic bodies were identified using 1,000x magnification using the Nikon 90i microscope ensemble described above. Images of mitotic *Drosophila S2* macrophage-like cells were used as a reference [36,52,59]. Mitotic cells were verified as hemocytes by morphology and by their phagocytic ability. Mitotic indices were calculated by determining the percentage of dividing hemocytes in each mosquito after inspecting 800 to 1,000 cells, and statistical analysis was performed by one-way ANOVA. Multinucleated cells with no apparent spindles were seen (a rare observation) but not counted as mitotic cells because they likely arise from cell fusion or abnormal mitosis [60,61]. To test whether the hemocyte collection methodology affected the results, proboscis snips [62] and low injection/recovery [14] hemocyte collection methods were also conducted. Comparison of these data to the data collected by standard perfusion verified that dividing cells can be observed using any hemocyte collection method.

## Abbreviations

ANOVA: analysis of variance; DIC: differential interference contrast; FBS: fetal bovine serum; GFP: green fluorescent protein; LB broth: Luria-Bertani’s rich nutrient medium.

## Competing interests

The authors declare that they have no competing interests.

## Authors’ contributions

JGK and JFH designed the study. JGK performed the experiments. JGK and JFH analyzed the data and wrote the manuscript. Both authors read and approved the final manuscript.
